# URGENT 1.5: diagnostic accuracy of the modified HEART score, with fingerstick point-of-care troponin testing, in ruling out acute coronary syndrome

**DOI:** 10.1007/s12471-021-01646-8

**Published:** 2021-11-24

**Authors:** L. H. Koper, L. D. S. Frenk, J. G. Meeder, F. H. M. van Osch, A. L. Bruinen, M. J. W. Janssen, A. W. J. van ’t Hof, B. M. Rahel

**Affiliations:** 1 Department of Cardiology, VieCuri Medical Centre, Venlo, The Netherlands; 2 Department of Clinical Epidemiology, VieCuri Medical Centre, Venlo, The Netherlands; 3 Department of Complex Genetics, School of Nutrition and Metabolism (NUTRIM), Maastricht University, Maastricht, The Netherlands; 4 Laboratory of Clinical Chemistry and Haematology, VieCuri Medical Centre, Venlo, The Netherlands; 5 Department of Cardiology, Maastricht University Medical Centre+, Maastricht, The Netherlands; 6 Department of Cardiology, Zuyderland MC, Heerlen, The Netherlands; 7 Cardiovascular Research Institute Maastricht (CARIM), Maastricht, The Netherlands

**Keywords:** Modified HEART score, Acute coronary syndrome, Point-of-care troponin testing, Fingerstick, Capillary blood

## Abstract

**Background:**

The HEART score is a validated risk stratification tool for chest pain patients presenting to the emergency department and was recently investigated for implementation in a pre-hospital setting. Fingerstick (capillary blood) point-of-care (POC) troponin testing enables quick measurements outside the hospital and seems easier to implement than the current venous blood sampling techniques. This study investigates the diagnostic accuracy of the modified HEART score, integrating fingerstick POC troponin testing, in ruling out acute coronary syndrome (ACS).

**Methods:**

The data of 96 patients with chest pain, included in a study investigating a novel POC troponin device under development at the cardiac emergency department, were analysed retrospectively. Based on the patients’ admission data and capillary POC high-sensitivity troponin I (hs-cTnI) results, the modified HEART score was determined. The outcome measure, for evaluating the diagnostic accuracy of the modified HEART score, was the occurrence of ACS.

**Results:**

Of the total study population, 33 patients (34%) were diagnosed with ACS. Seventeen patients (18%) were classified as low risk (0–3 points) and one patient (6%) in this group was diagnosed with ACS. The sensitivity and negative predictive value of the modified HEART score was 97.0 and 97.6%, respectively.

**Conclusion:**

The modified HEART score, integrating capillary POC hs-cTnI results, is a promising tool for ruling out ACS in patients with chest pain presenting to the cardiac emergency department. These results encourage prospective investigation into the integration of fingerstick POC troponin testing in the modified HEART score in a pre-hospital setting.

**Supplementary Information:**

The online version of this article (10.1007/s12471-021-01646-8) contains supplementary material, which is available to authorized users.

## What’s new?

- Fingerstick (capillary blood) point-of-care (POC) troponin testing seems easier to implement outside the hospital.
- This study found that the modified HEART score, integrating fingerstick POC high-sensitivity troponin I (hs-cTnI) testing, is a promising tool for ruling out acute coronary syndrome in low-risk patients with chest pain presenting to the cardiac emergency department.
- Risk stratification with the modified HEART score seems to be similar when integrating capillary POC hs-cTnI results versus standard venous high-sensitivity troponin T results.
- This study encourages a prospective investigation into the application of the modified HEART score, integrating fingerstick POC hs-cTnI testing, in a pre-hospital setting.

## Introduction

The (original) HEART score [1] is a validated risk stratification tool for patients with chest pain admitted to the emergency department (ED). Based on the five elements of the HEART score (i.e. history, electrocardiogram, age, risk factors and troponin level), patients receive a total score between zero and ten points, which represents the risk of a major adverse cardiac event (MACE): i.e. acute myocardial infarction, all-cause death, percutaneous coronary intervention or coronary artery bypass grafting [1, 2].

Although the HEART score is validated for use in the ED, the FamouS Triage trial [2] showed that ruling out a myocardial infarction in a pre-hospital setting is safe in chest pain patients without ST-segment elevation. Ishak et al. integrated high-sensitivity troponin T results (hs-cTnT) into the HEART score, measured from venous blood drawn in the ambulance [2], and therefore defined it as the ‘modified HEART score’. Since fingerstick (capillary blood) point-of-care (POC) troponin testing enables rapid analyses outside the hospital, implementation of this method in a pre-hospital setting seems easier than the current venous blood sampling techniques [3]. On-site fingerstick POC troponin testing could provide an earlier diagnosis or exclusion of an acute coronary syndrome (ACS), resulting in increased accuracy and speed of referral [3]. This study evaluates the diagnostic accuracy of the modified HEART score, integrating fingerstick (capillary blood) POC troponin testing, in ruling out ACS in chest pain patients without ST-segment elevation myocardial infarction (NSTEMI) presenting to the cardiac ED.

## Methods

### Study design and population

This is a retrospective registry study. Statistical analysis was performed on data of patients participating in a novel POC troponin device study, between September 2019 and March 2020, at the cardiac ED of VieCuri Medical Centre. This study was a prospective, observational cohort study, investigating a novel POC device under development for high-sensitivity troponin I (hs-cTnI) testing and included capillary blood samples obtained by fingerstick. Additional information can be found at clinicaltrials.gov, NCT number: 04153006 (data to be published).

The study originally included patients 18 years of age and older, referred to the cardiac ED with chest pain and suspected ACS. Patients with out-of-hospital cardiac arrest, arrhythmia >110 beats/min, haemodynamic instability or suspicion of an acute non-coronary diagnosis, recent admission to a healthcare institution for the same set of symptoms or inability/refusal to provide informed consent were excluded from the study. Subsequently excluded from participation in/eligibility for the URGENT 1.5 trial (Table S1, Electronic Supplementary Material) were all ST-segment elevation acute myocardial infarction (STEMI) patients (*n* = 19), as were patients with missing capillary POC hs-cTnI results, due to device failure resulting from incorrect use (*n* = 6), POC hs-cTnI measurements not taken immediately upon arrival at the ED (*n* = 5) or falsely elevated POC hs-cTnI due to recent cardiac intervention (*n* = 2).

From all patients included in the study (*n* = 96), capillary blood samples (obtained by fingerstick) were collected. All patients received standard medical care at the discretion of the treating physician.

### Assessment of the modified HEART score

Patients’ medical files and admission data were used to determine the modified HEART score, based on the criteria shown in Tab. 1 [4]. Patients received a final score between zero and ten points and were assigned, on the basis of the original classification system [1, 5], to a low- (0–3 points), intermediate- (4–6 points) or high-risk (7–10 points) group. Two independent investigators assessed the elements and were blinded with regard to the final diagnosis and other elements. If there was disagreement, a third independent investigator assessed the element in order to reach a final conclusion.

**Table 1:** The five modified HEART score elements: history, electrocardiogram, age, risk factors and troponin level

Modified HEART score		
*H*istory	Highly suspicious	2
	Moderately suspicious	1
	Slightly suspicious	0
*E*lectrocardiogram	Significant ST-segment depression	2
	Non-specific repolarisation disturbance	1
	Normal	0
*A*ge	≥65 years	2
	>45–<65 years	1
	≤45 years	0
*R*isk factors^a^	≥3 risk factors or history of atherosclerotic disease^b^	2
	1 or 2 risk factors	1
	No risk factors known	0
*T*roponin	≥3 × normal limit	2
	>1–<3 × normal limit	1
	≤Normal limit	0

^a^Risk factors: diabetes, currently treated; smoker, current or recent <90 days; hypertension, diagnosed and/or treated; obesity: body mass index >30 kg/m^2^; hypercholesterolaemia, diagnosed and/or treated; family history of coronary artery disease

^b^Atherosclerotic disease: prior myocardial infarction or coronary revascularisation (e.g. percutaneous coronary intervention or coronary artery bypass grafting), stroke or peripheral artery disease

Detailed explanation of all the HEART score elements is found in the original HEART score validation studies by Backus et al. [4] and Six et al. [5]. In addition, in the URGENT 1.5 trial a positive family history was defined as a history of coronary artery disease in first-degree relatives <65 years. The definition of hypercholesterolaemia was expanded to include ‘and/or low-density lipoprotein >3.2 mmol/l in previous laboratory results’. The two-point element ‘electrocardiogram’ was expanded to include ‘and/or inverted T waves, appropriate for a subacute infarction, new or not known to be old’. Missing data were classified as negative or not present.

### Integrating POC troponin results

The 99th percentile upper reference limit (URL) value for the novel POC device under development for hs-cTnI was 18.5 ng/l for female and 27.1 ng/l for male patients and was used as the normal limit. In this study, the modified HEART score using the standard venous hs-cTnT results from routine laboratory testing was determined in addition. The 99th percentile URL value for hs-cTnT in the central laboratory (Cobas e 801, Roche Diagnostics) was 14 ng/l.

### Outcome measure

The final diagnosis, ACS or no ACS, was determined by the treating physician. ACS was defined as unstable angina and NSTEMI (STEMI patients were excluded). The treating physician based the diagnosis on the patients’ complaints, physical examination, electrocardiogram, laboratory results including venous hs-cTnT results and any additional imaging (e.g. coronary angiography). The occurrence of ACS among the low-, intermediate- and high-risk patients was recorded.

A secondary objective was the comparison of the modified HEART score including capillary POC hs-cTnI results versus standard venous hs-cTnT results. Additionally, the distribution of all the points assigned for the five elements of the modified HEART score was compared between the ACS and no ACS group.

### Statistical analysis

Statistical analysis was performed with IBM SPSS Statistics version 24. Patient characteristics at baseline were compared between the different risk groups. Continuous variables were expressed as mean ± standard deviation (SD) and differences between groups were assessed by the Student *t*-test or, in the case of subgroups, by one-way analysis of variance. Categorical data were expressed as number and percentage and the Pearson’s chi-square or Fisher’s exact test was used to assess differences between (sub)groups. The sensitivity, specificity, negative predictive value (NPV) and positive predictive value were calculated using an online calculator (https://www.medcalc.org/calc/diagnostic_test.php).

## Results

### Baseline characteristics

Patient characteristics are presented in Tab. 2. Of the 96 patients included in this study, 17 (18%) were classified as low risk (0–3 points), 60 (63%) as intermediate risk (4–6 points) and 19 (20%) as high risk (7–10 points). The mean age of the study population was 66.9 years (±12.5 SD) and 51 patients (53%) were male. The mean age, prevalence of hypertension and history of atherosclerotic disease were significantly different among the risk groups. In the total study cohort 33 patients (34%) were diagnosed with ACS and differed significantly between the low- and high-risk group: 1 (6%) versus 16 (84%).

**Table 2:** Patient characteristics at baseline (in the total study population and per risk group of the modified HEART score)

	All patients	Modified HEART score 0–3	Modified HEART score 4–6	Modified HEART score 7–10	*p-*value
Population, *n* (%)	96 (100)	17 (17.7)	60 (62.5)	19 (19.8)	
Age, mean (SD)	66.9 (±12.5)	58.7 (±12.0)	67.4 (±11.8)	73.3 (±11.7)	0.002
Male gender, *n* (%)	51 (53.1)	7 (41.2)	32 (53.3)	12 (63.2)	0.418
Diabetes, *n* (%)	7 (7.3)	1 (5.9)	3 (5)	3 (15.8)	0.269
Smoker, *n* (%)	20 (20.8)	6 (35.3)	8 (13.3)	6 (31.6)	0.058
Hypertension, *n* (%)	59 (61.5)	6 (35.3)	39 (65)	14 (73.7)	0.040
Obesity, *n* (%)	24 (25)	3 (17.6)	18 (30)	3 (15.8)	0.395
Hypercholesterolaemia, *n* (%)	80 (83.3)	12 (70.6)	51 (85)	17 (89.5)	0.333
Family history of coronary artery disease, *n* (%)	39 (40.6)	4 (23.5)	25 (41.7)	10 (52.6)	0.200
History of atherosclerotic disease, *n* (%)	47 (49)	2 (11.8)	32 (53.3)	13 (68.4)	0.002
Acute coronary syndrome, *n* (%)	33 (34.4)	1 (5.9)	16 (26.7)	16 (84.2)	<0.001

### Main outcome

In the total study cohort 33 patients (34%) were diagnosed with ACS. Of the 17 patients in the low-risk group, 1 (6%) was diagnosed with ACS. In the intermediate- and high-risk groups 16 out of 60 patients (27%) and 16 out of 19 patients (84%) were diagnosed with ACS. The mean POC troponin results in the low-, intermediate- and high-risk group were 3.65 ng/l, 47.7 ng/l and 266.1 ng/l, respectively.

The sensitivity and NPV of the modified HEART score for ruling out ACS in low-risk patients were 97.0 and 97.6%, respectively, as shown in Tab. 3.

**Table 3:** Sensitivity, specificity, positive predictive value and negative predictive value of the modified HEART score for ruling out acute coronary syndrome in low-risk patients

	Modified HEART score
Sensitivity, % (95% CI)	97.0% (84.2–99.9)
Specificity, % (95% CI)	25.4% (15.3–37.9)
Positive predictive value, % (95% CI)^a^	21.0% (18.6–23.7)
Negative predictive value, % (95% CI)^a^	97.6% (85.0–99.7)

*CI* confidence interval

^a^The negative and positive predictive value are based on an estimated prevalence of acute coronary syndrome of 17% found in the population of chest pain patients presenting to the cardiac emergency department in previous studies [1, 2]

### Capillary POC hs-cTnI versus venous hs-cTnT

In 24 patients (25%) a difference was found when comparing the modified HEART score based on capillary POC hs-cTnI results versus standard baseline venous hs-cTnT results. Of those 24 patients, 21 (88%) did not change to another risk category despite having a different modified HEART score. Of the total 96 patients, 3 (3%) changed risk groups: 2 from low risk to intermediate risk and 1 from intermediate risk to high risk. Low- versus intermediate/high-risk stratification corresponded in 98% (*n* = 94/96).

### Modified HEART score: ACS versus no ACS

The distribution of all points assigned for the five elements of the modified HEART score in the ACS and no ACS group is presented in Tab. 4. The modified HEART score differed significantly between the two groups for the elements history, electrocardiogram and POC troponin results. The average modified HEART score of the ACS versus no ACS group was 6.33 (±1.8) and 4.22 (±1.3), respectively, and differed significantly.

**Table 4:** The distribution of all points assigned for the five elements of the modified HEART score in the acute coronary syndrome (*ACS*) versus no ACS group

	No ACS *n* = 63			ACS *n* = 33			Fisher exact *p*-value
Assigned score	0	1	2	0	1	2	
History, *n* (%)	26 (41)	34 (54)	3 (5)	5 (15)	14 (42.5)	14 (42.5)	<0.001
ECG, *n* (%)	40 (63.5)	23 (36.5)	0 (0)	9 (27)	20 (61)	4 (12)	<0.001
Age, *n* (%)	2 (3)	29 (46)	32 (51)	2 (6)	9 (27)	22 (67)	0.150
Risk factors, *n* (%)	0 (0)	21 (33)	42 (67)	1 (3)	10 (30)	22 (67)	0.510
POC troponin results, *n* (%)	58 (92)	5 (8)	0 (0)	15 (46)	4 (12)	14 (42)	<0.001
Modified HEART score, mean (SD)		4.22 (1.3)			6.33 (1.8)		<0.001

*ECG* electrocardiogram, *POC* point-of-care, *SD* standard deviation

## Discussion

This retrospective registry study on the integration of fingerstick (capillary blood) POC hs-cTnI testing into the modified HEART score shows promising results and encourages further prospective investigation in larger patient groups. The sensitivity and the NPV of the modified HEART score in this study are high (Tab. 3). These results are comparable with the sensitivity (96%) and NPV (95%) of the original HEART score found in a trial by Wang et al. [6]. By using the modified HEART score, including only one POC troponin measurement, ACS could be ruled out with a high degree of certainty in 17% (*n* = 16/96) of the total study population and in 94% (*n* = 16/17) of the low-risk patients.

In addition, the modified HEART score based on capillary POC hs-cTnI results versus standard venous hs-cTnT results showed a 25% difference. However, low- versus intermediate-/high-risk stratification was similar in 98%. These findings are comparable to those in the study by van Dongen et al. [7], which compared the HEART score based on POC troponin T versus hs-cTnT and found a different HEART score among 19% and different risk classification in low versus intermediate/high risk in only 2%. Another study by Aldous et al. [8] found similar results, showing the diagnostic accuracy of POC troponin I versus hs-cTnI results to be equal. These findings demonstrate that risk stratification according to the modified HEART score integrating capillary POC hs-cTnI results is reliable.

Evaluating the study population of the URGENT 1.5 trial, we found 18% of the patients to be classified as low risk and a diagnosis of ACS in this group in one patient (6%). These results do not entirely match those of the validation study of the original HEART score by Backus et al. [1] and the FamouS Triage trial [2], which observed higher proportions of patients in the low-risk group (36% in both studies) and lower occurrence of MACE (1.7 and 0%, respectively). These differences might be the result of the smaller study population. In addition, the outcome measure, diagnosis of ACS, is not completely similar to the definition of MACE in the above-mentioned studies. MACE does not include unstable angina in contrast to ACS, but includes death by any cause [1, 2]. Moreover, by including unstable angina, the proportion of patients in the ACS group that score low in the POC troponin element increases, since unstable angina is considered to be present in patients with ischaemic symptoms suggestive of an ACS but no elevation in troponin. Furthermore, in contrast to the validation study by Backus et al. [1], the distribution of the assigned points among the elements age and risk factors of the modified HEART score did not differ significantly between the ACS and no ACS group (Tab. 4). This could be due to the low number of patients under the age of 45 years (4%) and a single patient (1%) without risk factors. Our study population was also of higher age and had a higher prevalence of hypercholesterolaemia. The higher prevalence of hypercholesterolaemia could be the result of the difference in definition between the studies.

Since fingerstick POC troponin testing seems favourable and easy to implement outside the hospital, the modified HEART score could be a reliable tool for risk stratification in a pre-hospital setting. For implementation of the modified HEART score in a pre-hospital setting, skilled healthcare providers and good knowledge of the extensive modified HEART score criteria, as described by Backus et al. [4] and Six et al. [5], are required. A feasibility study by Mol et al. (reported in Chap. 8 of the doctoral thesis of K. Mol [9]) showed that the implementation of the HEART score among general practitioners is feasible and safe and thereby encourages further prospective investigation in a pre-hospital setting.

### Limitations

Because of the retrospective character there are several limitations to this study. Assessment of the modified HEART score was based on the (admission) data available in the medical files, but not all necessary information to score the different elements was available for every patient. In addition, the elements history and ECG were more sensitive to a subjective assessment. This limitation was addressed by involving at least two independent assessors. A second limitation of this study was the diagnosis of ACS. There were no predefined criteria for the diagnosis of ACS, making the final diagnosis, taken from the medical file, dependent on the assessment of the physician involved at admission. Additionally, the study included only 96 patients, and is therefore smaller in size than other studies involving the HEART score. Selection bias could be expected because in 6 months only 96 patients were included and presentation of chest pain patients to the cardiac ED is much higher. Since this is a single-centre study, the data may not be fully generalisable.

## Conclusion and recommendations

The modified HEART score, integrating fingerstick (capillary blood) POC hs-cTnI testing, is a promising tool for risk stratification and seems to be safe to rule out ACS in low-risk chest pain patients presenting to the cardiac ED. The sensitivity and NPV of the modified HEART score was 97.0 and 97.6%, respectively. Since fingerstick POC troponin testing seems favourable and easy to implement outside the hospital, the modified HEART score could be a reliable tool for risk stratification in a pre-hospital setting and could thereby reduce unnecessary admissions and associated healthcare costs [2]. These results encourage further prospective investigation into the applicability of the modified HEART score, integrating fingerstick POC troponin testing, in a pre-hospital setting.

## Supplementary Information

Table S1 Inclusion and exclusion criteria of the URGENT 1.5 trial
