# Supplementary material for: URGENT 1.5: diagnostic accuracy of the modified HEART score, with fingerstick point-of-care troponin testing, in ruling out acute coronary syndrome
Source: Neth Heart J. 2021 Nov 24;30(7-8):360–9. doi: 10.1007/s12471-021-01646-8 (PMC9270546; doi:10.1007/s12471-021-01646-8)
Supplement: Supplementary file 1 — Table S1 Inclusion and exclusion criteria of the URGENT 1.5 trial [file 12471_2021_1646_MOESM1_ESM.docx]

**Table S1** Inclusion and exclusion criteria of the URGENT 1.5 trial. *ED* emergency department, *ACS* acute coronary syndrome, *STEMI* ST-segment elevation acute myocardial infarction, *POC hs-cTnI* point-of-care high-sensitivity cardiac troponin I, *PCI* percutaneous coronary intervention

**Starting population: 128 patients**

| **Inclusion criteria** | **Exclusion criteria** |
| --- | --- |
| - Age 18 years or older - Referred to cardiac ED with chest pain suspected of ACS; inclusion on arrival | - Out-of-hospital cardiac arrest - Sudden-onset tachycardia and a frequency of 110 beats/min or higher (supraventricular or ventricular) - Haemodynamically unstable patients or suspicion of an acute non-coronary diagnosis, e.g. pulmonary embolism, thoracic aortic dissection etc. - Recent admission to a healthcare institution for the same set of symptoms before being transferred to the participating clinical site - Not willing or not able to provide informed consent due to their medical condition as judged by the physician - STEMI patients - Patients with missing capillary POC hs-cTnI results, due to device failure resulting from incorrect use - POC hs-cTnI measurements not taken immediately upon arrival at the ED - Falsely elevated POC hs-cTnI due to recent cardiac intervention (e.g. PCI) |

**96 inclusions URGENT 1.5**
